# RELATIONSHIP BETWEEN 5 EPIGENETIC CLOCKS, TELOMERE LENGTH, AND FUNCTIONAL CAPACITY ASSESSED IN OLDER ADULTS: CROSS-SECTIONAL AND LONGITUDINAL ANALYSES

**DOI:** 10.1093/geroni/igad104.0065

**Published:** 2023-12-21

**Authors:** Ilja Demuth

**Affiliations:** Charité – Universitätsmedizin Berlin, Berlin, Berlin, Germany

## Abstract

DOI: 10.1093/gerona/glab381

